# Supplementary material for: Ultra-Low DNA Input into Whole Genome Methylation Assays and Detection of Oncogenic Methylation and Copy Number Variants in Circulating Tumour DNA
Source: Epigenomes. 2021 Feb 19;5(1):6. doi: 10.3390/epigenomes5010006 (PMC7610445; doi:10.3390/epigenomes5010006)

Supplementary figure 3: (A) Multi-dimensional scaling plot of blood (green), ctDNA (orange) and fresh tissues (purple) demonstrating seperation of groups; (B) Hierarchical clustering plot showing seperate clustering of Blood,ctDNA and fresh tissue groups

(A)

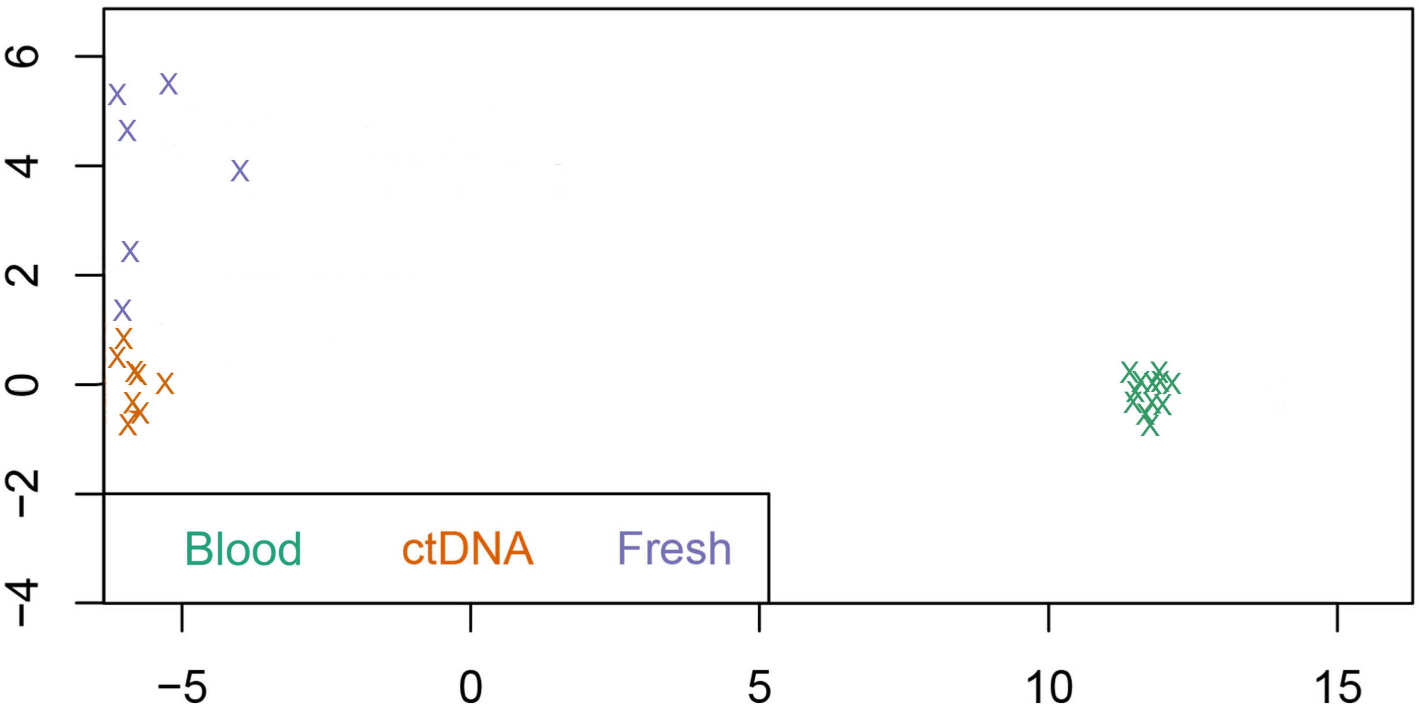

(B)

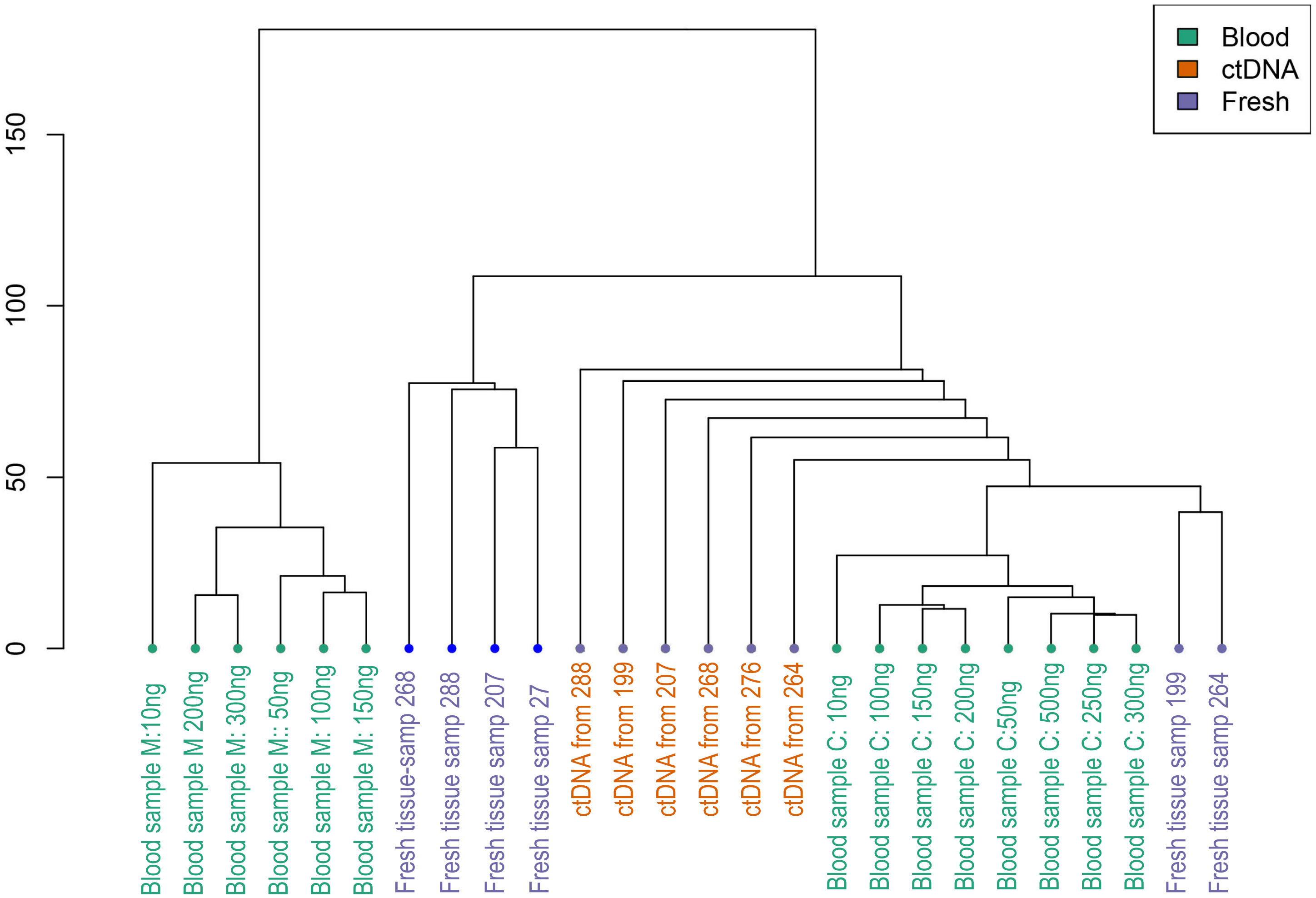

Supplement: Supplementary file 1 [file epigenomes-05-00006-s001.zip › Suppl final/Supplementary Figure S3.pdf]
