# Supplementary material for: Ultra-Low DNA Input into Whole Genome Methylation Assays and Detection of Oncogenic Methylation and Copy Number Variants in Circulating Tumour DNA
Source: Epigenomes. 2021 Feb 19;5(1):6. doi: 10.3390/epigenomes5010006 (PMC7610445; doi:10.3390/epigenomes5010006)

Supplementary figure 4: Density plot of percentage methylation (beta) vs. density in all tissue types

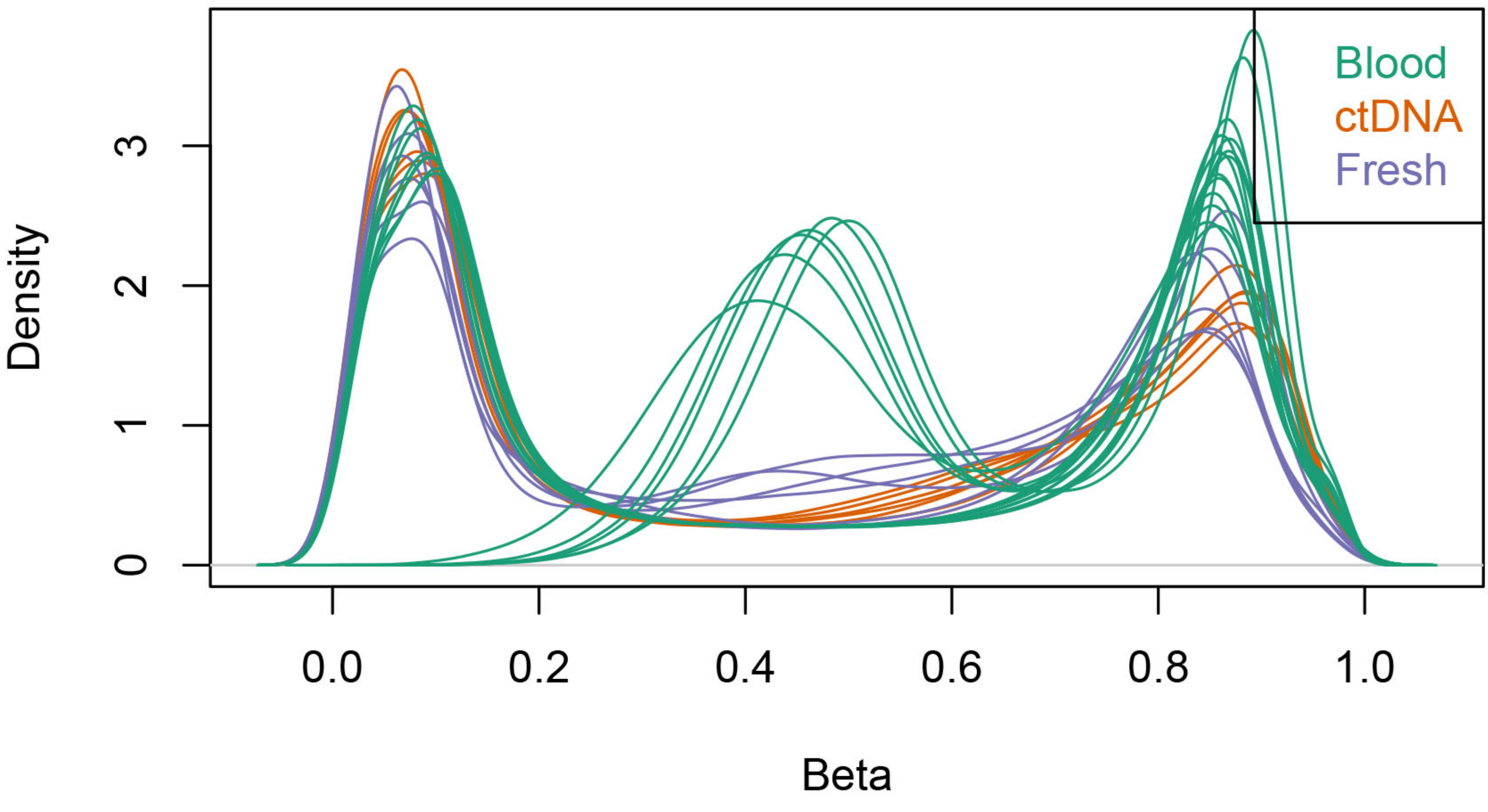

Supplement: Supplementary file 1 [file epigenomes-05-00006-s001.zip › Suppl final/Supplementary Figure S4.pdf]
