# Supplementary figures and images for: Ultra-Low DNA Input into Whole Genome Methylation Assays and Detection of Oncogenic Methylation and Copy Number Variants in Circulating Tumour DNA
Source: Epigenomes. 2021 Feb 19;5(1):6. doi: 10.3390/epigenomes5010006 (PMC7610445; doi:10.3390/epigenomes5010006)

# Low input experiments

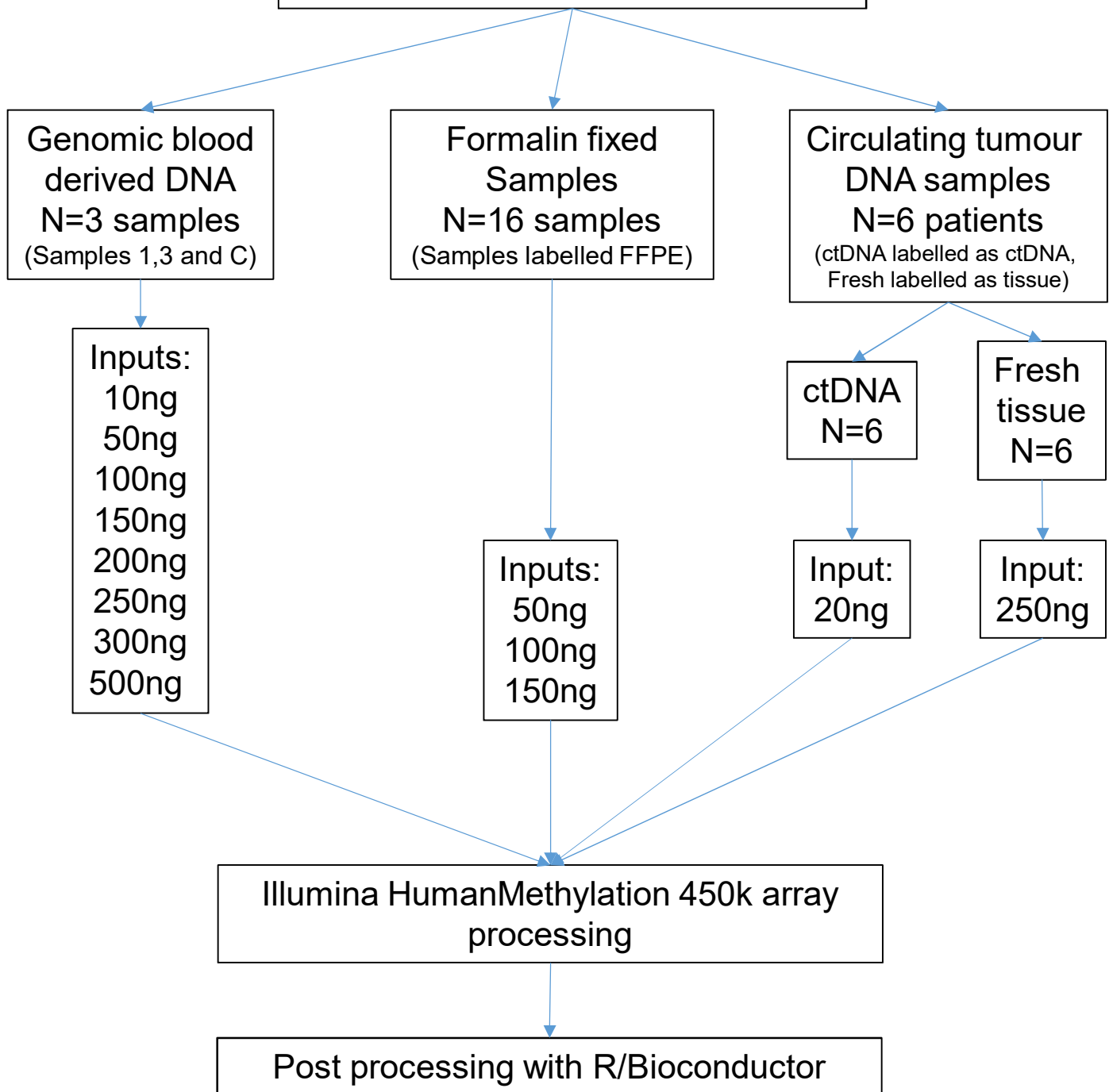

Supplement: Supplementary file 1 [file epigenomes-05-00006-s001.zip › Suppl final/Supplementary Figure S1.pdf]

## Difference vs. average: Bland-Altman of ctDNA vs. fresh tissue

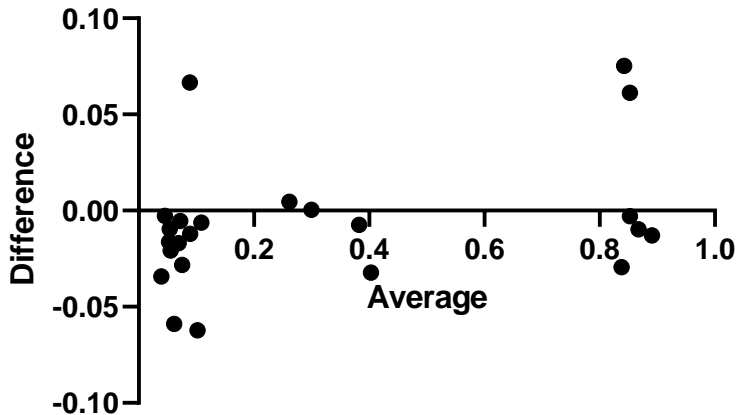

Supplement: Supplementary file 1 [file epigenomes-05-00006-s001.zip › Suppl final/Supplementary Figure S2.pdf]

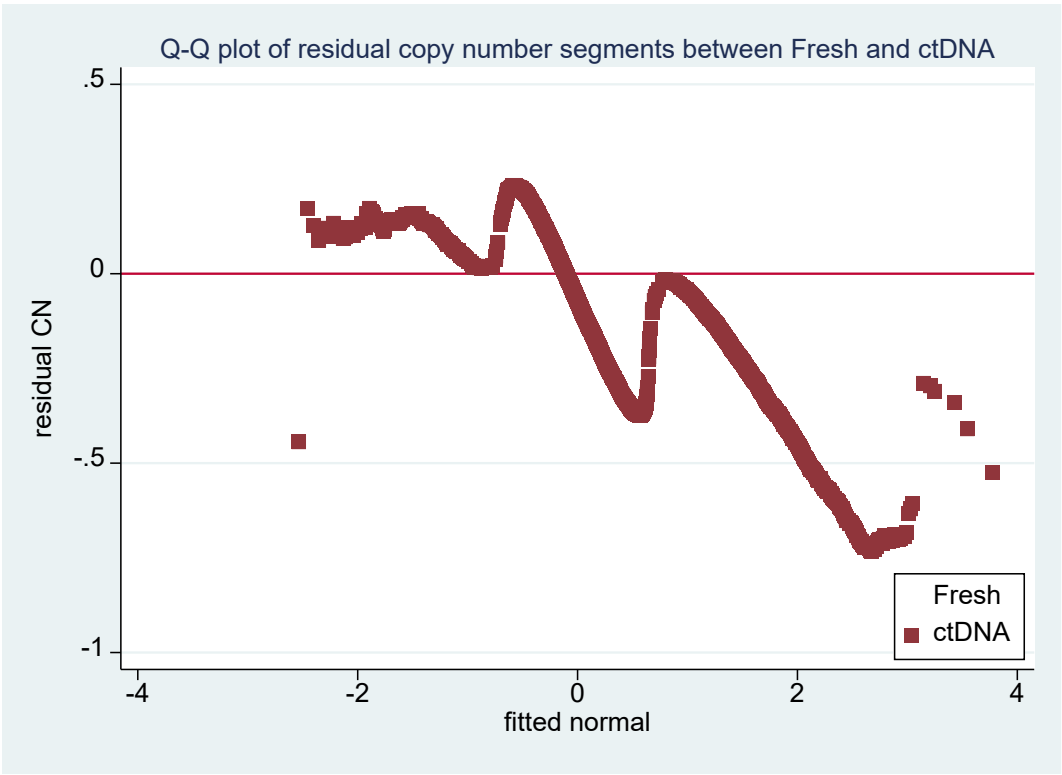

Supplement: Supplementary file 1 [file epigenomes-05-00006-s001.zip › Suppl final/Supplementary Figure S5.pdf]
